# Atrial Fibrillation Detection from Wrist Photoplethysmography Signals Using Smartwatches

**DOI:** 10.1038/s41598-019-49092-2

**Published:** 2019-10-21

**Authors:** Syed Khairul Bashar, Dong Han, Shirin Hajeb-Mohammadalipour, Eric Ding, Cody Whitcomb, David D. McManus, Ki H. Chon

**Affiliations:** 1 Department of Biomedical Engineering, University of Connecticut, Storrs, CT USA; 2 Division of Cardiology, University of Massachusetts Medical School, Worcester, MA USA

**Keywords:** Cardiac device therapy, Biomedical engineering

## Abstract

Detection of atrial fibrillation (AF) from a wrist watch photoplethysmogram (PPG) signal is important because the wrist watch form factor enables long term continuous monitoring of arrhythmia in an easy and non-invasive manner. We have developed a novel method not only to detect AF from a smart wrist watch PPG signal, but also to determine whether the recorded PPG signal is corrupted by motion artifacts or not. We detect motion and noise artifacts based on the accelerometer signal and variable frequency complex demodulation based time-frequency analysis of the PPG signal. After that, we use the root mean square of successive differences and sample entropy, calculated from the beat-to-beat intervals of the PPG signal, to distinguish AF from normal rhythm. We then use a premature atrial contraction detection algorithm to have more accurate AF identification and to reduce false alarms. Two separate datasets have been used in this study to test the efficacy of the proposed method, which shows a combined sensitivity, specificity and accuracy of 98.18%, 97.43% and 97.54% across the datasets.

## Introduction

Atrial fibrillation (AF) is the most common sustained arrhythmia and is associated with significant morbidity and mortality^1^. AF is often undiagnosed, affects about 34 million people worldwide, and has an estimated prevalence of 3% in the adult population^2,3^. AF also increases the risk of stroke by a factor of 4 to 5. It is responsible for about 15% of all ischemic strokes and causes a 2-fold increased risk of heart disease related death^4,5^. Accurate detection of silent AF and long term monitoring can prevent stroke significantly, if it leads to well-established AF treatment with medications such as prophylactic anticoagulants^6^.

There are numerous studies of AF detection from electrocardiogram (ECG) signals. These algorithms are based on either variability of RR intervals^7,8^ or detection of the absence of a p-wave in the ECG^9,10^. However, with advancements in miniaturization of device technologies, wearable health monitoring is gaining attraction. Since photoplethysmography (PPG) is a noninvasive technique and can be recorded on most modern devices, research has been focused on implementing PPG-based AF detection as an alternative to ECG-based solutions^11^. Video PPG signals recorded from smartphone cameras can be used to detect AF as shown in^12^. In^13^, authors present an 8-layer deep neural network which can passively detect AF from a PPG signal obtained with a commercially available smartwatch. Both irregularity of heart rhythm and absence of p-wave detection methods have been implemented in^14^, in which the authors use a Kardia band to record both the ECG and PPG signals from a smartwatch and use it to classify AF from normal sinus rhythm. In^15^, the authors have extracted both PPG time series features and spectral features via wavelet analysis along with a convolutional neural network and built an Elastic Net logistic model to classify AF and non-AF. Most recently, in^16^, AF was discriminated from sinus rhythm using complex nonlinear combination analysis of pulse intervals preceded by a data quality check, although the measurements were performed while the subjects were sitting in a comfortable position in a quiet hospital environment. Moreover, none of these smartwatch-based AF detection methods dealt with premature atrial contraction (PAC) and premature ventricular contraction (PVC) rhythms, which when present can cause false positive detection of AF.

For the neural network-based methods (i.e., deep learning), hundreds to thousands of parameters have to be learned, which may not be practical for a wearable device. Also for the wrist ECG-based system, one needs to physically touch the ECG lead with a finger on the non-watch wearing hand to close the circuit, thus preventing continuous recording. Another major challenge for PPG-based wearable systems is motion artifacts. Traditional PPG noise detection methods are for the fingertip, forehead and ear PPG. One method relies on statistical measures like skewness and kurtosis with phase coupling, described in^17^, while Shannon entropy along with Renyi’s entropy is used in^18^ to quantify a PPG signal quality index. In^19^, a method based on time-frequency based features and a support vector machine has been described for the forehead PPG signal. Motion artifact detection for smartphone video PPG has been described in^20^ which can detect spike-type artifacts as commonly found in phone PPG signals. However, for wristwatch PPG signals, it is not clear if any of these noise detection methods are applicable and effective.

To combat the challenges of AF detection with a smartwatch, in this study we present a novel method based on the wrist PPG signal for AF detection, which also accounts for motion and noise artifacts (MNA). After discarding the MNA-corrupted PPG segments with a newly developed MNA detection algorithm, we calculate features to classify AF from normal sinus rhythm (NSR). Moreover, as a two-step verification, we also look for premature atrial contraction (PAC) patterns to ensure a more robust identification. We examined the efficacy of our approaches using two different datasets.

## Dataset Description

**Figure 1:**
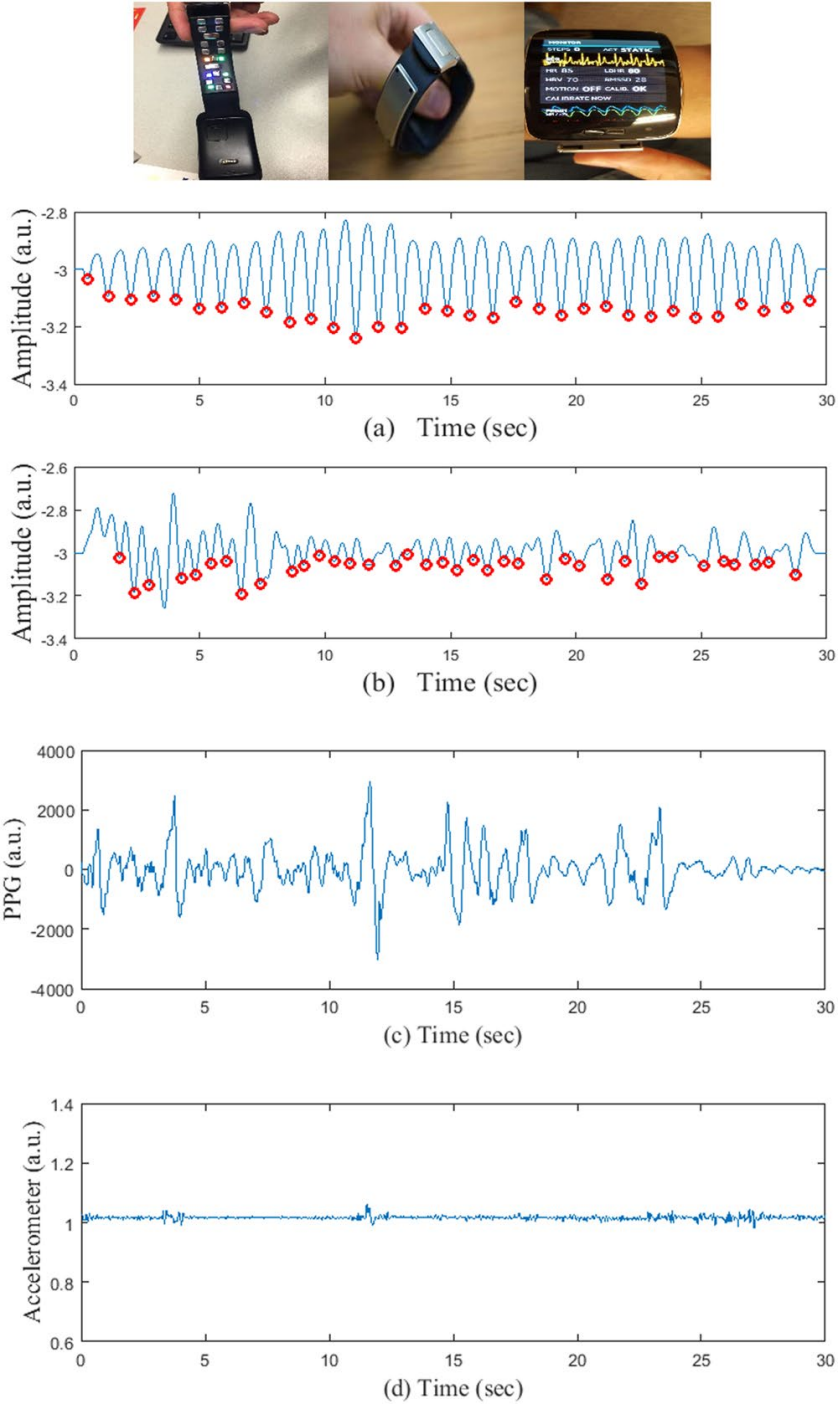
(Top row)- Left: wristband of the Simband with LEDs for PPG; Center: wristband ECG; Right: Simband display of PPG and ECG. Panel (a) a sample 30-sec clean and panel (b) corrupted PPG segment, respectively, from Simband. Panel (c) a corrupted 30-sec PPG signal when the *ACC* is clean and panel (d) the corresponding *ACC* signal.

In this study, two different data sets have been used. The UMass database has been collected at the University of Massachusetts Medical School while the “Chonlab database” has been collected at the Chon lab at the University of Connecticut. All experiments were performed in accordance with relevant guidelines and regulations provided by the respective institutional review boards. For both of the experiments, the PPG signal has been recorded from a smart wristwatch provided by Samsung (also known as “Simband”). The Simband has 8 PPG sensors, an accelerometer with 3 axes, an ECG lead, a temperature sensor and other sensors (Fig. 1). Details can be found in^21^.

### UMass database

Data collection protocol was approved by the University of Massachusetts Medical School (UMass) Institutional Review Board (IRB). The UMass database consists of 37 subjects, 10 of them having AF. Subjects were instructed to perform several kinds of movements to replicate daily movements. Participants were approached following their ambulatory clinic appointment and taken to a free exam room where they gave their informed consent to participate in the study and received a detailed outline of the study procedure. The next step involved the fitting of the Simband and a 7-lead Holter monitor (Rozinn RZ153+ Series, Rozinn Electronics Inc., USA). The Holter monitor’s ECG data were used as the reference. Participants were taken through a standardized protocol consisting of the following activities: 2 minutes of completely sitting still while placing their thumb on the ECG sensor of the Simband, 2 minutes of slow walking (approximately 2 mph), 30 seconds of rest while standing still, 2 minutes of fast walking (approximately 4 mph), 1 minute of rest, 1 minute of up and down arm movement, 1 minute of random wrist movement, 30 seconds of rest while standing, 1 minute of alternating sitting and standing, 2 minutes of going up and down a set of stairs, and 1 minute of deep breathing^22^. Details about the study population for the UMass database can be found in^23^.

### Chonlab database

The data collection protocol was approved by the University of Connecticut’s Institutional Review Board (IRB). This dataset was recorded in the Chon Lab (University of Connecticut) from 9 healthy male subjects with normal sinus rhythm. Informed consent was obtained from all subjects before the data collection. For each subject, the PPG signal was recorded from the left wrist using the Simband. Simultaneously, an ECG signal (collected from the chest using a Holter monitor) was used to calculate the reference heart rate. The total experimental protocol lasted for 16 minutes; during the data recording subjects performed several activities similar to daily movements. The activities included: sitting completely still for 3 minutes on a chair, slowly walking on a treadmill for 3 minutes (at 2 mph), jogging/fast walking for 3 minutes (at 4 mph), randomly moving both hands for 2 minutes, bending to lift a sand bag and then placing it back on a specific location several times for 3 minutes, and finally, sitting still again on a chair for 2 minutes as the resting period.

For both data sets, the sampling frequency of the Simband’s PPG and accelerometer was 128 Hz while for the Holter monitor, it was 180 Hz (later downsampled to 128 Hz).

## Proposed Method

The proposed method consists of first preprocessing the PPG signal followed by detecting motion and noise artifacts (MNA). Next, from the segments of PPG signals determined to be clean, heart rate is determined by peak detection. From the heart rate fluctuations, classification is made between AF and normal sinus rhythm.

### Preprocessing of the acquired data

In our case, the recorded PPG signals were first divided into 30-second non-overlapping windows. The rationale behind choosing 30 seconds is that we wanted to analyze whether our AF detection method can work on such short data segments. For each 30-second window, data were filtered with a bandpass filter (6^th^ order Butterworth bandpass with cut off frequencies of 0.5 to 20 Hz). PPG and accelerometer signals were down-sampled to 50 Hz and 30 Hz, respectively. Next, both the PPG and accelerometer signals were converted to zero mean, of unity variance. For the accelerometer (*ACC*) signal, the magnitude was calculated by combining the data from three axes of the accelerometer as *ACC* = $\sqrt{({a}_{x}^{2}+{a}_{y}^{2}+{a}_{z}^{2})}$ where *a*_*x*_, *a*_*y*_ and *a*_*z*_ are the axes. The appropriately pre-processed PPG and *ACC* segments should then be fed into the next phase of the algorithm.

### Motion and noise artifact detection

When there is extensive movement, the PPG signal from a smartwatch is affected by MNA. If the PPG is corrupted with MNA, then it will give rise to incorrect peak detection which can eventually lead to false detection of AF. Figure 1(a,b) show representative clean and corrupted PPG segments of 30 seconds.

To avoid incorrect peak detection due to MNA, a preprocessed PPG segment is first checked to determine whether it is corrupted or not. For this purpose, both the PPG and the accelerometer signals have been used. It is obvious that when there is any motion or movement, the magnitude of the accelerometer changes significantly. To utilize the change in the *ACC*, we calculated the mean of the *ACC* signal after transforming the signal to unit variance. We observed that when there is enough movement/motion on the accelerometer, the variance increases and there is a change in the accelerometer magnitude. Since the *ACC* is of unity variance, as the variance increases due to motion, the mean value of *ACC* becomes lower for a corrupted segment while the mean value is higher for a clean segment. A threshold (*ACC*_*TH*_) has been applied to this mean to detect the accelerometer motion.

However, using only the accelerometer signal to determine motion and noise artifact is not sufficient, for many reasons. One of the main problems is that the PPG signal quality can be poor even without arm movement. This can occur due to a myriad of reasons but two primary reasons are bad contact of the PPG sensor to the skin (watch not worn tightly), and darker skin color in some subjects. Thus, without analyzing the signal quality and solely relying on the accelerometer data, one risks analyzing MNA-corrupted data, which will most likely lead to false AF detection. Figure 1(c) shows a sample PPG segment where the *ACC* is clean (Fig. 1(d)). However, as is evident from Fig. 1(a), the PPG signal is badly distorted.

### Variable frequency complex demodulation (VFCDM) based noise detection

To detect PPG waveform distortion, time domain features are not that effective because a small change in the PPG time series can lead to huge deviation in the time domain based features. As a result, in this study, PPG signal quality has been analyzed in the time-frequency domain. Based on the analysis of the time-frequency spectra (TFS), we want to determine whether the time domain PPG signal is corrupted by MNA or not.

**Figure 2:**
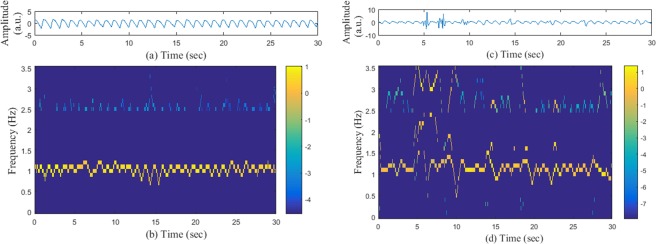
(**b**) TFS (log scale) of the clean PPG segment shown in (**a**). (**d**) TFS (log scale) of the corrupted PPG segment of (**c**).

To calculate the TFS, the variable frequency complex demodulation (VFCDM) method has been implemented. VFCDM is shown to provide high time-frequency resolution while retaining the amplitude distribution of the signal. It offers a significant improvement in computation time, as only the center frequencies are considered in the analysis. Details of the VFCDM can be found in^24^. After subtracting the mean and making the input PPG signal of unity variance, VFCDM is applied to a 30 second PPG segment. Figure 2(b) shows the TFS (only for the heart rate range) obtained from a clean PPG (shown in Fig. 2(a)) by using VFCDM, while Fig. 2(d) shows the similar TFS for a corrupted PPG segment (shown in Fig. 2(c)).

In the heart rate (HR) range, the dominant frequency components (high energy components) are continuous for the clean PPG segment and the TFS is relatively “clean.” However, when there is noise present in the data or when the PPG is distorted by MNA, there is significant change in the TFS. The distortion creates high frequency harmonics with relatively high amplitudes and the dominant frequency trace (i.e., HR trace) becomes discontinuous (see Fig. 2(d)). As a result, the TFS becomes “noisy.” We have used these properties to make distinctions between clean and corrupted PPG segments.

For a clean PPG segment in the HR range, the dominant frequency components (up to two largest peaks) may contain significant energy and the other frequency components possess negligible energy (zero, for the ideal case). To quantify this property, from the TFS in the HR range, we define two quantities corresponding to “clean signal energy (*Q*_1_)” and “noise energy (*Q*_2_)” as follows:

*Q*_1_ = energy of the first two dominant frequency components.

*Q*_2_ = energy of the rest of the frequency components in the HR range.

For a clean signal, we want to have high *Q*_1_ values and very low (ideally zero) *Q*_2_ values for the majority of the time (i.e., for most of the time instances). By counting the time instances of *Q*_1_ and *Q*_2_, we can separate corrupted PPG segments from clean PPG segments. We have used the *ACC* signal as the override factor for the noise detection algorithm, which means, if the *ACC* is found to be noisy (noisier than a certain threshold *ACC*_*TH*_), then we trust the accelerometer data and give the MNA detection result by overriding the VFCDM based MNA detection method.

**Figure 3:**
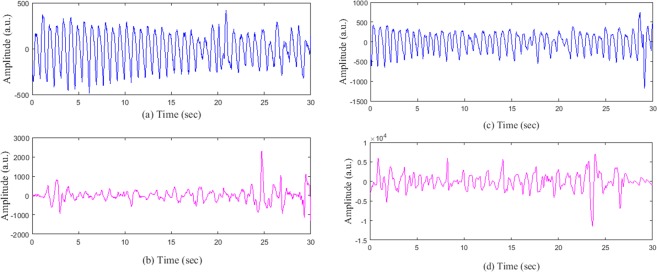
(**a**) A clean PPG segment (NSR) which has been detected as clean by the proposed MNA detection algorithm; (**b**) a corrupted PPG segment which has been detected as corrupted; (**c**) a clean PPG segment (AF) which has been detected as clean by the proposed MNA detection algorithm; (**d**) a corrupted PPG segment which has been detected as corrupted.

Next, we show representative examples of the MNA detection algorithm for both AF and NSR subjects. Figure 3(a,b) show the MNA detection output for the normal sinus rhythm while Fig. 3(c,d) are for AF PPG segments. The proposed method can correctly detect Fig. 3(a,c) as clean segments while Fig. 3(b,d) are determined to be corrupted. This shows the effectiveness of our method for both AF and NSR subjects.

### Atrial fibrillation detection

Once a PPG segment is detected as clean, the next phase is to detect whether it is an AF segment or not. AF simply refers to irregularly irregular heart rate. In other words, AF is a random sequence of heart beat intervals with increased beat-to-beat variability and complexity^8^. The peak-to-peak distances in the PPG waveform during an AF interlude are highly irregular, which results in widely varying heart rates. To calculate the HR, a PPG peak detection algorithm based on local peak and envelope detection has been used here.

To use this randomness and complexity as the discriminating property of AF, we have calculated the root mean square of successive differences (RMSSD) and sample entropy from the pulse intervals corresponding to each 30-sec PPG segment.

RMSSD is a statistical measure of a time series and for the pulse intervals, it can estimate the beat-to-beat variability. The RMSSD value is expected to be higher in AF subjects than in NSR subjects, as AF exhibits higher variability than do regular rhythms^8^. The RMSSD value has been divided by the mean of the RR time series as it is more consistent than using RMSSD of the time series alone, because the variability and mean of HR values differ from subject to subject and segment to segment.

Sample Entropy (*SampEn*) is a measure of randomness and has been widely used in the literature to determine the irregularity of a signal/time series. Sample entropy provides the complexity measure and has been used in arrhythmia detection for ECG and video PPG signals^25^.

**Figure 4:**
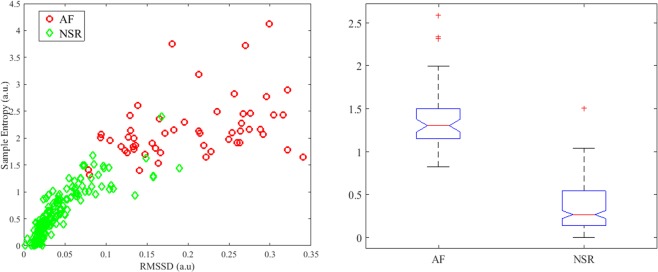
(Left) Scatter plot for RMSSD and Sample entropy obtained from AF and NSR segments; (Right) Box plot of the combined feature for AF and NSR segments.

Figure 4 shows the scatter plot for the RMSSD and *SampEn* values obtained from both NSR and AF segments of the UMass data. The green diamond markers represent the NSR data whereas the red circles represent the AF segments. From the scatter plot, it is evident that RMSSD and *SampEn* are excellent parameters for classifying AF and NSR. However, instead of using two different thresholds with a logic combination to discriminate AF and NSR segments, a new feature has been formed by taking a weighted average of the RMSSD and *SampEn*. The new combined feature (“*Comb*”) is denoted by

1 $$Comb=w\times RMSSD+(1-w)\times SampEn$$

Once the combined feature has been formed, a simple threshold has been applied to classify the AF and NSR.

2 $$If\,Comb\ge 0.94,\,decision=AF.$$

$$Otherwise,\,decision=NSR.$$

Figure 4 also shows the box plots of the combined feature for the two classes. From the figure, it can be clearly observed that the medians are non-overlapping, which means that the proposed feature has good discrimination properties.

To calculate the statistical significance, a two-sided Wilcoxon rank sum test has been performed, as the sample sizes are different for the two classes and features do not follow normal distribution. The resulting p-value was 4.7065*e*^−28^, which rejects the null hypothesis that data from the two classes are samples from continuous distributions with equal medians.

### PAC/PVC detection

**Figure 5:**
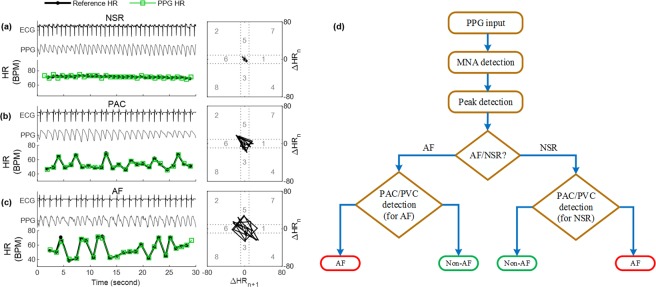
Sample 30-sec ECG, PPG and HR data with corresponding Poincaré plots for (**a**) NSR, (**b**) PAC and (**c**) AF subjects. In (**a**–**c**), top plot line shows the ECG, middle plot line shows the PPG and the bottom plot line shows the HR in BPM where black and green lines are the reference and PPG HR, respectively. (**d**) Flow chart of the proposed AF vs. non-AF classification algorithm.

In certain cases, when the subject has premature atrial contractions (PAC) or premature ventricular contractions (PVC), their pulse intervals have more variation, similar to AF. As a result, there are more variations in the HR than in a typical NSR segment. This extra variation in HR can make a PAC segment look like an AF segment, which would be a false positive detection. PAC/PVC is not AF (it is a regularly irregular change of HR) and for this study, our goal is to classify AF vs. non-AF PPG segments. Thus, once a segment is initially detected as AF or non-AF, the next step is to check for whether it has PAC/PVC patterns present in the pulse intervals or not. To detect the PAC/PVC patterns, Poincare plots derived from the R-R intervals have been used in our method^26^. For the PAC/PVC segments, due to the repeated PAC/PVC beats, triangular kite-shaped patterns are found in the Poincare plot trajectory of the difference of the HR as shown in Fig. 5(b). However, for the ideal NSR segments, almost all the patterns are inside the center quadrat while for the AF segments, there is no distinct parents as shown in Fig. 5(a,c), respectively. It is to be noted that, in the PAC/PVC detection step, we also do a second level AF screening. Figure 5(d) shows the complete flow diagram of the proposed AF vs. non-AF classification scheme. PAC/PVC patterns are included in both AF and non-AF decisions.

## Experimental Results

### Result on UMass and chonlab data sets

The UMass database contains 37 subjects, including 10 AF subjects. Since the experimental protocol consists of several exercise tasks, a vast portion of the recorded PPG signal is corrupted with MNA. From the UMass database, we have 2,394 30-second PPG segments obtained from the 37 subjects. Among those segments, our MNA detection algorithm determined that 314 segments were clean and these were used for the AF vs. NSR classification. Out of the 314 clean-detected segments, 55 are from the AF subjects while 259 are from the NSR subjects.

As the first step, our AF detection algorithm classified 52 segments as AF (out of 55) and 248 as NSR (out of 259).

Hence, the confusion matrix for the AF vs. NSR classification is as shown in Table 1, where TP means true positive, TN means true negative, FP means false positive, and FN means false negative. The sensitivity, specificity and accuracy have been calculated in the following way:

3 $$\begin{array}{rcl}{\rm{Sensitivity}} & = & {\rm{TP}}/({\rm{TP}}+{\rm{FN}})\\ {\rm{Sensitivity}} & = & {\rm{TN}}/({\rm{TN}}+{\rm{FP}})\\ {\rm{Sensitivity}} & = & {\rm{TN}}/({\rm{TN}}+{\rm{FP}})\end{array}$$

**Table 1:** AF vs NSR classification performance results.

	Without PAC/PVC algorithm				With PAC/PVC algorithm		
	Predicted Class				Predicted Class		
**UMass Data Results**							
True class		AF	NSR	True class		AF	NSR
	AF	52 (TP)	3 (FN)		AF	54 (TP)	1 (FN)
	NSR	11 (FP)	248 (TN)		NSR	5 (FP)	254 (TN)
**Results for Both Data sets**							
True Class		AF	NSR	True Class		AF	NSR
	AF	52 (TP)	3 (FN)		AF	54 (TP)	1 (FN)
	NSR	18 (FP)	293 (TN)		NSR	8 (FP)	303 (TN)

The sensitivity, specificity and accuracy are 94.55%, 95.75% and 95.54%, respectively.

Although the sensitivity and specificity are quite satisfactory, we still have 11 false positives and most importantly, there are 3 missed detections of AF (false negatives). Some false alarms are due to the PAC/PVC beats, as they introduce variability in the heart rate. As a result, to reduce the number of false alarms, as the second step we apply the PAC detection algorithm. Our hope is that the PAC/PVC detection algorithm can not only reduce some false alarms by detecting the PAC/PVC patterns, but can also improve the AF detection by analyzing the Poincaré trajectory. By doing the two-step algorithm, we reduced the 6 false positives and correctly detected 2 more AF segments. As a result, the improved sensitivity, specificity and accuracy of our algorithm becomes 98.18%, 98.07% and 98.09%. In Table 1, “UMass Data Results: with PAC/PVC algorithm” presents the revised confusion matrix. It is to be noted that the PAC/PVC algorithm successfully detected 6 out of 7 PAC/PVC segments which caused false positives in our initial analysis.

**Figure 6:**
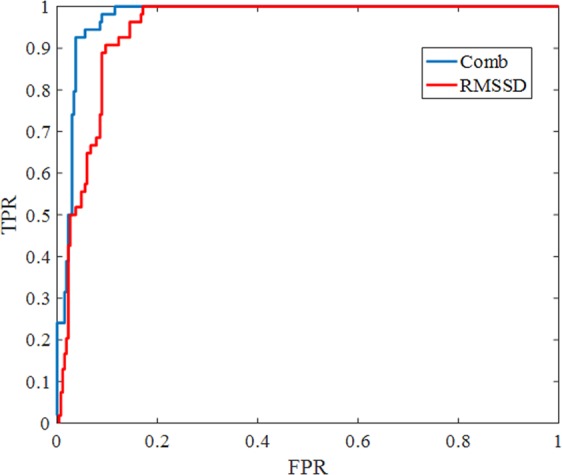
ROC curve analysis of the UMass dataset.

To show the classification performance, we provide the receiver operating characteristic (ROC) curve for the UMass database in Fig. 6. FPR indicates false positive rate and TPR is the true positive rate. The “blue curve” is for the “Comb” value (with w = 0.4) while the “red curve” is for the RMSSD alone. The area under the ROC curve for Comb is 0.9748 which is much better than 0.9465 (for the RMSSD).

For the Chonlab database, we have only NSR subjects (9 subjects; 285 segments). As a result, with the help of this database, we can test the specificity of the proposed method. After we ran the MNA detection algorithm, it determined that 52 30-second segments were clean from 285 total available segments. At first, the AF detection algorithm resulted in 45 true negatives and 7 false positives, but with the help of the PAC/PVC pattern detection algorithm, 4 false AF detections were revised to be NSR. Thus, the final number of TN and FP for the Chonlab database is 49 and 3, respectively, resulting in 49/52 = 94.23% specificity. In Table 1, “Results for both data sets” gives the combined confusion matrices for all the clean segments across two different data sets (i.e., for a total of 366 30-second PPG segments) using both approaches (i.e., without the PAC/PVC detection step and with the PAC/PVC detection step).

Overall, using the tabulated values reported in Table 1, without the PAC/PVC detection step, the sensitivity, specificity and accuracy are found to be 94.55%, 94.21% and 94.26%, respectively. However, with the PAC/PVC detection algorithm, the values become 98.18%, 97.43% and 97.54%, respectively, which demonstrate the significance of the proposed method.

### Analysis of noise effect on the algorithm

For the UMass database, visual adjudication of clean PPG segments was performed; we found 292 segments to be clean. Our MNA detection algorithm identified 289 of these clean segments correctly. Table 2: “effect of noise on the algorithm” shows the confusion matrix using only the adjudicated clean data for both with and without PAC/PVC detection. For the clean adjudicated data, with the PAC/PVC detection, the proposed method achieved 98.15% sensitivity, 99.16% specificity, and 98.97% accuracy. These results indicate that, if clean PPG segments can be provided, our proposed algorithm can produce more accurate detection of AF, by a few percent, than can our automated MNA detection approach.

**Table 2:** Effect of Noise on the Algorithm.

	Without PAC/PVC algorithm					With PAC/PVC algorithm		
	Predicted Class					Predicted Class		
**Results from UMass Data (only clean adjudicated)**								
True Class		AF	NSR		True Class		AF	NSR
	AF	51 (TP)	3 (FN)			AF	53 (TP)	1 (FN)
	NSR	7 (FP)	231 (TN)			NSR	2 (FP)	236 (TN)
**Comparison of False positives for different approaches**								
		**Method type**	**No of total segments**	**Segments determined as clean**	**False positives**	**Portion of false positives (%)**		
		No MNA detection	908	908	488	53.74		
		Only *ACC* detection	908	349	110	31.52		
		Proposed MNA	908	134	3	2.24		

Finally, to demonstrate the impact of the MNA detection algorithm, we present the analysis based on 12 NSR subjects from the UMass dataset containing 908 30-second PPG segments. Our assumption is that, since the motion and noise artifacts can lead to inaccurate estimation of HR, it may lead to false positives for the NSR subjects. So this analysis is limited to NSR subjects only. In Table 2, “Comparison of False positives for different approaches” shows the comparison of three methods (without any MNA detection, with MNA detection based on the accelerometer only, and with the proposed MNA detection) in terms of false positives and the percentage of false positives out of the total segments calculated as clean. From the table, it can be seen that using only the *ACC* gives 110 false positives while the proposed method gives only 3 false positives. Although only 134 segments pass through the AF detection scheme after we use the proposed MNA detection algorithm, the false positive percent is only 2.24%, which is significantly lower than the 31.52% found when using *ACC* only. This clearly shows that the proposed MNA detection algorithm can significantly reduce the number of false positives caused by the noise and motion artifacts.

## Discussion

The major contribution of this study is to propose a complete scheme for smartwatch-based arrhythmia detection including MNA detection, peak detection, and AF vs. non-AF classification with PAC/PVC pattern recognition. We are one of the first to develop a comprehensive time-frequency based PPG artifact detection criteria for smartwatch. With this MNA detection algorithm, we reduced the percentage of false positives to a great extent by discarding the corrupted PPG segments. We also showed that using only the accelerometer data for MNA detection would still result in a lot of misdetections, thus reflecting the need to adopt PPG waveform quality based detection, which is incorporated in our proposed MNA detection method. Although a large portion of the PPG data was discarded due to the MNA algorithm, this was necessary to reduce false AF detection due to poor signal quality. The fact that this MNA detection can be performed automatically in real time proves the usefulness of this approach in continuous AF monitoring while doing daily activities.

Our method is a completely deterministic approach whereas previous studies’ methods are based on deep learning^13,15^. While deep-learning models can work on a remote server with powerful processors, they are not suitable for a smartwatch’s microprocessor. Moreover, our experimental setup differs from those of the previous studies. Data collection for previous studies using smartwatches was largely confined to clinical environments or to an immobile sitting position^11,13,15,16^ whereas in our study, subjects were allowed to move extensively while performing typical daily activities like walking, moving their arms, lifting weights, and the like. As a result, our study is more representative of real life scenarios. In addition, we have embedded our AF detection algorithm into a Samsung Gear S3, illustrating that our method is implementable within the memory limits of the watch and is real-time realizable.

For the AF vs. non-AF decision, the study proposed a two-step detection with PAC/PVC pattern recognition. The derived features showed significant discriminatory properties. Moreover, with the PAC detection step, not only could we reduce false positives, but also we recovered two more true positives by finding the irregular trajectories in the Poincaré plot. To the best of our knowledge, we are one of the first to consider discrimination of PAC/PVC rhythms from smartwatch PPG signals.

One limitation of this study is the low signal coverage due to the MNA algorithm; the number of segments with good signal quality is rather low when subjects are moving. However, we expect most of the good quality data will be acquired when subjects are sleeping or sitting still, for example watching TV or reading. In addition, since the prevalence of AF occurs in subjects over age 60, with more than 60-80% of AF occurring in subjects who are over 70, we would expect less motion artifact contaminated data for this elderly population. Certainly, it is clear from our work that whether statistical methods as we have employed or machine learning algorithms are used, to be able to accurately detect AF and discriminate PAC/PVC from NSR and AF, it is imperative that subjects remain stationary during recording. This is certainly the main limitation of PPG for AF detection from smartwatches, but given that it can be used for continuous monitoring, it is our opinion that there will be sufficient periods (especially during sleep and sitting) when good PPG signals can be obtained from elderly subjects. As demonstrated in our paper, once we have good quality PPG data, the accuracy and reliability of AF detection are quite excellent. Future work may include reconstruction of the PPG signal from corrupted segments so that we can use some of the less noisy PPG segments, thus leading to better signal coverage.

## Conclusion

In this study, a novel method has been proposed to detect AF from a smartwatch-based PPG signal which takes motion and noise artifacts into consideration. Using a time-frequency based MNA detection method along with the accelerometer signal shows great promise for continuous AF monitoring. The AF detection method presented impressive performance, which was even enhanced by applying a second-level PAC/PVC pattern detection method. The high sensitivity, specificity, and accuracy obtained from both datasets, proved the efficacy of the complete AF discrimination method and its potential for real life implementation.
